# A249 PROTON PUMP INHIBITOR USE AND RISK OF DEMENTIA: AN UMBRELLA REVIEW OF SYSTEMATIC REVIEWS AND META-ANALYSES

**DOI:** 10.1093/jcag/gwae059.249

**Published:** 2025-02-10

**Authors:** B E Abu-Mallouh, K Al-Naamani, M Martel, A Barkun

**Affiliations:** Medicine, Jordan University of Science and Technology, Irbid, Jordan; Department of Medicine, Armed Forces Hospital, Muscat, Oman; McGill University Health Center Research Institute, Montreal, Canada, Montreal, QC, Canada; Division of Gastroenterology, McGill University, Montreal, Canada, Montreal, QC, Canada

## Abstract

**Background:**

Clinical studies about the association between long term PPI use and dementia have been inconsistent, with systematic reviews and meta-analyses (SR/MA) reaching disparate conclusions.

**Aims:**

This umbrella review of SR/MA attempts to clarify the current understanding of any possible association linking PPI use to dementia.

**Methods:**

We searched Embase, Medline, CENTRAL and ISI Web of Science for fully published SR/MA in English up to June 2024 using controlled vocabulary and text words ((1) proton pump and (2) dementia (dementia and mild cognitive impairment, Alzheimer disease)). SR/MA methodological quality was scored using the AMSTAR-2 tool.

**Results:**

Eleven SR/MA published (May 2016 - February 2023) described 21 studies (one randomized trial). Only one high-quality SR/MA significantly associated PPI use with dementia. None of six SR/MA investigating PPI use and Alzheimer’s disease showed a significant association. Significant associations with dementia in subgroup analyses were found in one SR/MA each for older patients (age > 65 years) (HR=1.39 (1.17;1.65)), long term PPI use (> 5 years) (HR=1.28 (1.12; 1.46)), short term PPI use (< 5 years) (RR=1.62 (1.40–1.86)), when only considering higher quality studies (RR=1.54 (1.12; 2.13)), and amongst European populations (HR=1.46 (1.23–1.73)). Two SR/MA found an association when only assessing prospective cohort studies (RR=1.36 (1.19; 1.55)), (RR=1.44 (1.36; 1.52)).

**Conclusions:**

Most SR/MA suggest no link between PPI use and dementia. Only properly designed, adequately powered randomized trials can rule out a small but significant association possibly linking PPI use to dementia.

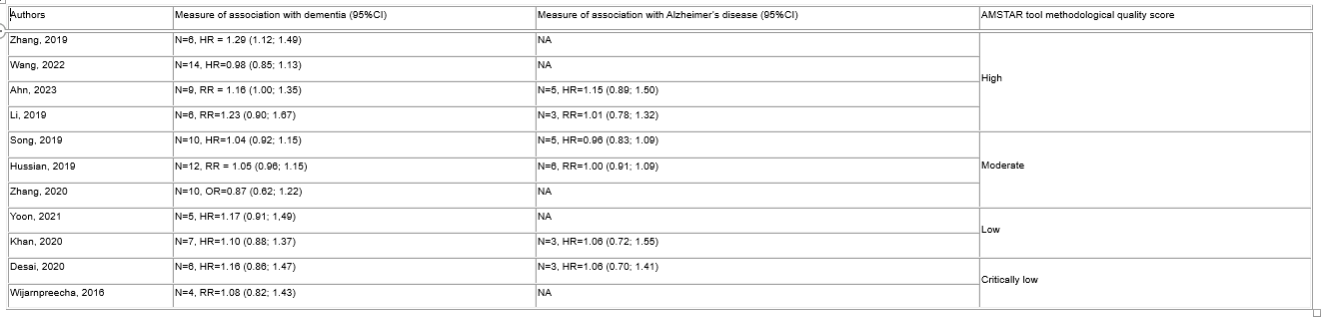

N = study number in SR/MA, HR = hazards ratio, RR = relative risk, OR = odds ratio, NA = Not applicable

**Funding Agencies:**

None

